# Usefulness of the anterior transpetrosal approach for pontine cavernous malformations

**DOI:** 10.3171/2019.7.FocusVid.19125

**Published:** 2019-07-01

**Authors:** Hiroki Morisako, Takeo Goto, Christian A. Bohoun, Hironori Arima, Tsutomu Ichinose, Kenji Ohata

**Affiliations:** Department of Neurosurgery, Osaka City University Graduate School of Medicine, Abeno-ku, Osaka, Japan

**Keywords:** pontine cavernous malformation, surgical resection, anterior transpetrosal approach, video

## Abstract

Surgical resection of pontine cavernous malformation remains a particularly formidable challenge. We report the surgical outcome of eight cases with pontine cavernous malformations operated using the anterior transpetrosal approach. All cases presented with neurological deficits caused by hemorrhage before surgery. Gross-total removal was achieved in all cases without any postoperative complication such as worsening of facial nerve palsy, ocular movement disorder, or hemiplegia. A small incision of the pons with multidirectional dissection is the most important factor for minimizing postoperative neurological deficits, so resection of a pontine cavernous malformation via this approach can be an alternative better option.

The video can be found here: https://youtu.be/2Q2CUhBbo28.

## Transcript

0:35 We report here our experiences in treating patients with pontine cavernous malformation via an anterior transpetrosal approach.

0:56 Surgical resection of pontine cavernous malformation remains a particularly formidable challenge in the neurosurgical field because of their deep-seated and eloquent locations. A small cortical incision with multidirectional dissection is the most important factor for minimizing postoperative neurological deficits.

0:56 Three entry zones can be used via an anterior transpetrosal approach to excise pontine lesions. On this illustration of the surgical view during a right anterior transpetrosal approach, three blue lines represent the entry zone to the pons. We can obtain a wide surgical corridor from the ventral and lateral side of the pons.

1:18 This 37-year-old male had a past surgical history of left midbrain cavernous malformation which was resected using a left lateral infratentorial supracerebellar approach. Preoperative images demonstrated a newly formed pontine cavernous malformation in the right pons.

1:37 He presented the left oculomotor nerve palsy and loss of right deep sensation related to the previous left midbrain cavernous malformation, and new deficits such as a right oculomotor nerve, right abducens nerve palsy, left hemiparesis, and truncal ataxia due to the new right pontine cavernous malformation. The pyramidal tract was compressed on the ventral medial side of the pons by the lesion.

2:03 The virtual simulation via a trans–fourth ventricular approach showed detailed microsurgical anatomy. The right medial lemniscus was located posteriolaterally to the cavernous malformation. There was also a risk of injury of the nucleas of the sixth or seventh cranial nerve.

2:22 The virtual simulation via a right anterior transpetrosal approach demonstrated the small cortical incision at the anterolateral part of the pons allowed multidirection dissection while maintaining safe and sufficient distance to pyramidal tract and medial lemniscus.

2:40Surgical technique. The patient was placed in a supine lateral position, elevating the upper half body at a 30° angle and keeping the temporal surface parallel to the floor.

2:51 The skin incision began at the upper margin of the zygomatic arch anterior to the tragus, turned 2–3 cm above the ear, and then descended behind the posterior margin of the mastoid process.

3:04 After the skin flap was raised, a temporalis fascia pericranial flap with a pedicle of the sternocleidomastoid muscle was used for the dural reconstruction at the end of surgery to prevent postoperative CSF leakage.

3:20 For the temporal craniotomy, burr holes were made at six different points, the asterion, the intersection of the supramastoid crest with the squamous suture, above the transverse sinus, the root of the zygoma, and the anterior and posterior aspects of the temporal bone. The outer cortical bone of the mastoid portion of the temporal bone was removed.

3:41 In the middle fossa, the dura mater over the temporal base was gently reflected to fully expose the entire course of the petrous ridge and apex by cutting the middle meningeal artery and dissecting the greater superficial petrosal nerve. After the dural dissection was complete, bony work encompassing anterior petrosectomy was performed. During drilling, care was taken to keep the membranous labyrinths of the semicircular canals intact for hearing preservation. Drilling of the petrous ridge is one of the key steps in obtaining a surgical corridor along its axis.

4:15 The middle fossa dura was opened along the inferior temporal lobe toward the superior petrosal sinus [SPS]. The subtemporal dural incision was made as far anterior as possible, and its medial extent ran along the lateral margin of Meckel’s cave. The SPS was divided by clips at a point anterior to the drainage point of the petrosal vein into the SPS to preserve its venous drainage. The presigmoid dura was opened along the drilled petrosal portion of the temporal bone as far anterior as possible, and the drainage of the petrosal vein into the SPS was inspected. The tentorium was cut completely to inspect the trochlear nerve and its entrance into the intradural part.

5:00 After superior retraction of the temporal lobe, the superior wall of Meckel’s cave was opened. The trigeminal nerve was mobilized laterally to expose the anterolateral part of the pons and increase the working space. After dissection of arachnoid membrane around the root exit zone of the trigeminal nerve, most suitable entry point of the pons was checked by using navigation system.

5:20 A small cortical incision was made at the anterolateral part of the pons and the cavernous malformation was exposed. At first fluid component of the cavernous malformation was evacuated and the margin was identified. The length of the corticotomy was about 5 mm. The viewing angle was changed from cranial-caudal to caudal-cranial for easier access. The lesion was gently removed piece by piece by multidirectional dissection.

6:20 A clear plane of separation between the cavernous malformation and the surface of pons was achieved with bipolar cautery and sharp microdissection. Finally, the lesion was removed completely.

7:00 All opened mastoid air cells were sealed with abdominal fat, and then the mastoid and petrous portions of the temporal bone and dural defect were entirely covered with the harvested temporal fascia pericranial flap, and cranioplasty was made.

7:16 The postoperative MR T2-weighted images showed gross-total resection of the lesion.

7:24 The eye movement disorders of this patient was recovered and facial nerve function was preserved completely.

7:30 From 2008 to 2018, eight cases of cavernous malformation were resected using an anterior transpetrosal approach at Osaka City University Hospital. Gross-total removal was achieved in all the cases.

7:44 All cases presented with neurological deficits caused by hemorrhage before surgery. No patients showed any postoperative complication such as worsening of facial nerve palsy, ocular movement disorder, or hemiparesis.

8:02 However, there is a contraindication of the anterior transpetrosal approach for pontine cavernous malformation. When venous anomalies connecting to the superior petrosal sinus exist, there is a high risk of damaging the venous flow during this procedure. This 49-year-old female had that venous anomalies, so we did not select our procedure.

8:28 In conclusion, a small cortical incision with multidirectional dissection is the most important factor for minimizing postoperative neurological deficits, so resection of pontine cavernous malformation via an anterior transpetrosal approach could be very useful.
